# Role for Arabidopsis *PLC7* in Stomatal Movement, Seed Mucilage Attachment, and Leaf Serration

**DOI:** 10.3389/fpls.2018.01721

**Published:** 2018-11-27

**Authors:** Ringo van Wijk, Qianqian Zhang, Xavier Zarza, Mart Lamers, Francisca Reyes Marquez, Aisha Guardia, Denise Scuffi, Carlos García-Mata, Wilco Ligterink, Michel A. Haring, Ana M. Laxalt, Teun Munnik

**Affiliations:** ^1^Section Plant Physiology, University of Amsterdam, Amsterdam, Netherlands; ^2^Section Plant Cell Biology, Swammerdam Institute for Life Sciences (SILS), University of Amsterdam, Amsterdam, Netherlands; ^3^Laboratory of Plant Physiology, Wageningen University and Research, Wageningen, Netherlands; ^4^Instituto de Investigaciones Biológicas (IIB-CONICET-UNMdP), Universidad Nacional de Mar del Plata, Mar del Plata, Argentina

**Keywords:** PLC, seed mucilage, leaf serration, ABA sensitivity, drought tolerance

## Abstract

Phospholipase C (PLC) has been suggested to play important roles in plant stress and development. To increase our understanding of PLC signaling in plants, we have started to analyze knock-out (KO), knock-down (KD) and overexpression mutants of *Arabidopsis thaliana*, which contains nine PLCs. Earlier, we characterized *PLC2, PLC3* and *PLC5*. Here, the role of *PLC7* is functionally addressed. Promoter-GUS analyses revealed that *PLC7* is specifically expressed in the phloem of roots, leaves and flowers, and is also present in trichomes and hydathodes. Two T-DNA insertion mutants were obtained, i.e., *plc7-3* being a KO- and *plc7-4* a KD line. In contrast to earlier characterized phloem-expressed *PLC* mutants, i.e., *plc3* and *plc5*, no defects in primary- or lateral root development were found for *plc7* mutants. Like *plc3* mutants, they were less sensitive to ABA during stomatal closure. Double-knockout *plc3 plc7* lines were lethal, but *plc5 plc7 (plc5/7)* double mutants were viable, and revealed several new phenotypes, not observed earlier in the single mutants. These include a defect in seed mucilage, enhanced leaf serration, and an increased tolerance to drought. Overexpression of *PLC7* enhanced drought tolerance too, similar to what was earlier found for *PLC3*-and *PLC5* overexpression. *In vivo* ^32^P_i_-labeling of seedlings and treatment with sorbitol to mimic drought stress, revealed stronger PIP_2_ responses in both drought-tolerant *plc5/7* and *PLC7*-*OE* mutants. Together, these results show novel functions for PLC in plant stress and development. Potential molecular mechanisms are discussed.

## Introduction

In animals, phospholipase C (PLC) plays a key role in the perception and transmission of extracellular signals into cells. Activated by extracellular receptors, PLC hydrolyses the membrane phospholipid, phosphatidylinositol 4,5-bisphosphate (PIP_2_) into two second messengers: inositol 1,4,5-trisphosphate (IP_3_) and diacylglycerol (DAG). While IP_3_ triggers the release of Ca^2+^ from the ER via a ligand-gated Ca^2+^ channel, the DAG remains in the plasma membrane where it recruitsand activates members of the protein kinase C (PKC) family and stimulates TRP- (Transient Receptor Potential-) channels. Subsequent increases in Ca^2+^ and phosphorylation status affects various protein targets that regulate multiple processes within and between cells (Irvine, 2006; Michell, 2008; Balla, 2013).

In plants, the PLC-signaling pathway is still enigmatic. Plants lack the primary targets for both IP_3_ and DAG, and have limited amounts of PIP_2_ in their membranes (van Leeuwen et al., 2007; Munnik and Nielsen, 2011; Munnik and Zarza, 2013; Munnik, 2014; Heilmann, 2016a,b; Gerth et al., 2017; Noack and Jaillais, 2017). Likely, they use phosphatidylinositol 4-phosphate (PIP) as an additional substrate and phosphorylate the resulting inositol phosphates and DAG into inositol polyphosphates (IPPs; e.g., IP_6_) and phosphatidic acid (PA), respectively, which can function as second messengers (Munnik, 2001; Gillaspy, 2013; Munnik, 2014; Heilmann, 2016a,b; Hou et al., 2016; Yao and Xue, 2018). IP_6_ levels increase upon ABA and release Ca^2+^ in guard cells to induce stomatal closure (Lemtiri-Chlieh et al., 2000, 2003). PA has also been implicated to play a role in ABA signaling, e.g., inhibiting ABI1 (Mishra et al., 2006), activating SnRK2 (Testerink et al., 2004; McLoughlin et al., 2012; Julkowska et al., 2015), and regulating ion channels (Camoni et al., 2012; McLoughlin and Testerink, 2013; Hite et al., 2014). Meanwhile, various other functions for IP_6_ and additional IPPs have been emerging that could be signaling downstream of PLC, including the pyro-phosphorylated IP_7_ and IP_8_ (Laha et al., 2015, 2016). In yeast and mammalian cells, IPP molecules play important roles in various nuclear processes, including gene transcription, chromatin remodeling, mRNA export and DNA repair, involving a wide range of cellular processes, such as osmoregulation, phosphate homeostasis, vesicular trafficking, apoptosis, cell cycle regulation, and ribosome synthesis (Monserrate and York, 2010; Thota and Bhandari, 2015; Williams et al., 2015). In plants, IP_6_ binds the auxin receptor, TIR1 (Tan et al., 2007), which is proposed to functionally regulate the SCF^TIR1^ ubiquitin-ligase complex to control downstream auxin mediated-gene expression (Leyser, 2018). Similarly, COI1, the receptor for jasmonate signaling binds IP_5_ (Sheard et al., 2010) or the pyrophosphorylated form of IP_5_, i.e., PP-IP_5_ (= IP_7_) (Laha et al., 2015, 2016), with functional significance for plant immunity (Mosblech et al., 2008, 2011; Murphy et al., 2008). GLE1, an mRNA export factor, has been identified as an IP_6_ target in Arabidopsis P_i_ homeostasis (Lee et al., 2015). SPX domain-containing proteins bind IPPs, including IP_6_, and many of these proteins are involved in P_i_ signaling (Kuo et al., 2014, 2018; Puga et al., 2014; Wild et al., 2016). For PA, several plant targets have been identified over the years, including protein kinases, proteins phosphatases, small G-proteins, RBOH (NADPH oxidase), GAPDH, ion channels and actin-binding proteins and PA has been implicated to regulate many cellular processes, including vesicular trafficking, cytoskeleton dynamics, and ion-channels (Munnik, 2001; Wang et al., 2006; Li et al., 2009, 2012; Pleskot et al., 2010, 2017; Testerink and Munnik, 2011; Thomas and Staiger, 2014; Hou et al., 2016; Ufer et al., 2017; Pokotylo et al., 2018; Yao and Xue, 2018). PA is not only generated via PLC and DAG kinase (DGK); it can also be formed via other DAG-generating enzymes, like non-specific PLCs (NPC), or directly, through phospholipase D (PLD) hydrolysis of structural phospholipids (Arisz et al., 2009; Munnik and Testerink, 2009; Pokotylo et al., 2013, 2018; Hou et al., 2016; Yao and Xue, 2018).

How, when, where and whether PLC signaling is involved in generating PA and IPPs is still largely unknown. Hence, tools to genetically manipulate PLC levels would be helpful to functionally address this. As such, silencing of *PLC* has revealed its importance in plant defense in tomato and Arabidopsis (Vossen et al., 2010; D’Ambrosio et al., 2017), in cytokinins- and gravity signaling in *Physcomitrella* (Repp et al., 2004), and in ABA signaling and stomatal control in tobacco and Arabidopsis (Sanchez and Chua, 2001; Hunt et al., 2003; Mills et al., 2004). In petunia and tobacco, PLC has been shown to regulate the tip growth of pollen tubes (Dowd et al., 2006; Helling et al., 2006). Arabidopsis T-DNA insertion mutants for *PLC3*, *PLC5* and *PLC9* have revealed roles for PLC in seed germination, primary- and lateral root development, ABA signaling and heat stress tolerance (Zheng et al., 2012; Gao et al., 2014; Zhang et al., 2018a,b), while *PLC2*-insertion mutants showed defects in female gametogenesis and embryo development (Li et al., 2015; Di Fino et al., 2017). Overexpression of *PLC* has been shown to increase the drought tolerance of maize, canola, tobacco and Arabidopsis (Wang et al., 2008; Georges et al., 2009; Tripathy et al., 2011; Zhang et al., 2018a,b). While it is still not clear how PLC exactly achieves all this, it is important that such molecular tools become available for further physiological- and biochemical analyses.

The Arabidopsis genome encodes 9 *PLC* genes, which are subdivided into four clades (Hunt et al., 2004; Tasma et al., 2008; Munnik, 2014; Pokotylo et al., 2014). Earlier, we found that knock-out (KO) mutants of *PLC3* and a knock-down (KD) mutant of *PLC5*, exhibited small defects in root development (Zhang et al., 2018a,b). Interestingly, *plc3plc5-*double mutants did not intensify the phenotype, even though they belong to different clades, indicating that another *PLC* could be involved. Since *PLC3* and *PLC5* were both specifically expressed in phloem-companion cells and revealed a “segmented” root-expression pattern from which lateral roots emerge, we searched for additional Arabidopsis *PLCs* that are phloem-specific and might explain the lack of additional effects on the double mutant. This resulted in the identification of *PLC7*, which again belongs to another clade. Here, the functional analysis of this PLC is described, revealing novel phenotypes and, hence function.

## Materials and Methods

### Plant Material

*Arabidopsis thaliana* (*Col-0*) was used throughout. The two T-DNA insertion mutants, *plc7-3* (SALK_030333) and *plc7-4* (SALK_148821) were obtained from the SALK collection ^1^. Homozygous plants were identified by PCR in F2 generation using gene-specific primers. For the identification of *plc7-3*, we used forward primer 5′-GATTTGGGTGATAAAGAAGTTTGG-3′; reverse primer 5′-CTCCACACAATCTCAGCATTAC-3′ and left border primer LBb1.3 (5′-ATTTTGCCGATTTCGGAAC-3′, in combination with the forward primer). For *plc7-4* identification, forward primer 5′-TCCTTCCTGTTATCCATGACG-3′; reverse primer 5′-TTGAAGAAAGCATCAAGGTGG-3′) and left border primer LBb1.3 (in combination with the reverse primer) were used. To generate a *plc5/7-*double mutant, *plc7-3* was crossed with *plc5-1* (SALK_144469), a T-DNA insertion KD mutant that was functionally complemented (Zhang et al., 2018b).

### Root Growth

Seeds were surface sterilized in a desiccator using 20 ml thin bleach and 1ml 37% HCl for 3 h, and then sowed on square petri dishes containing 30 ml of ½ strength of Murashige and Skoog (½MS) medium (pH 5.8), 0.5% sucrose, and 1.2% Daishin agar (Duchefa Biochemie). Plates were stratified at 4°C in the dark for 2 days, and then transferred to long day conditions (22°C, 16 h of light and 8h of dark) in a vertical position, under an angle of 70°. Four-day-old seedlings of comparable size were then transferred to ½MS-agar plates without sucrose and allowed to grow further for another 6–8 days. Plates were then scanned with an Epson Perfection V700 scanner and primary root length, lateral root number and average lateral root length from each genotype determined through ImageJ software (National Institutes of Health).

### Cloning and Plant Transformation

To generate *pPLC7::GUS-SYFP* reporter line, the *PLC7* promoter was amplified from genomic DNA using the following primers: PLC7proH3fw (5′-CCCAAGCTTGATCCTATCAATATTCCTAATTCAGC-3′) and PLC7proNheIrev (5′-CTAGCTAGCTTGAACAATTCCTCAAGTG-3′). The PCR product was cloned into pGEM-T easy and sequenced. A *Hind*III-pPLC7-*Nhe*I fragment was then ligated into pJV-GUS-SYFP, cut with *Hind*III and *Nhe*I. A *pPLC7::GUS-SYFP* fragment, cut with *Not*I and transferred to pGreenII-0229. A MultiSite Gateway Three-Fragment Vector Construction Kit ^2^ was used to generate *PLC7*-overexpression lines, driven by the *UBQ10* promotor (*pUBQ10::PLC7*). Oligonucleotide primers (5′-GGGGACAACGTTTGTACAAAAAAGCAGGCTATGTCGAAGCAAACATACAAAGT-3′ and 5′-GGGGACCACTTTGTACAAGAAAGCTGGGTCACAAACTCCAACCGCACAAGAA-3′) including attB1 and attB2 sites, were used to PCR *PLC7* from cDNA and was cloned into the donor vector (pDONR207) by using BP Clonase II enzyme mix to create entry clone BOX2. BOX1 was *pGEM-pUBQ10* entry clone containing attL4 and attR1 sites. BOX3 was pGEM-TNOS entry clone containing attR2 and attL3 sites. The three entry clones (BOX1, BOX2 and BOX3) and a destination vector (pGreen0125) were used in MultiSite Gateway LR recombination reaction to create the expression clone (Invitrogen).

All constructs were transformed into *Agrobacterium tumefaciens*, strain GV3101, which was subsequently used to transform Arabidopsis plants by floral dip (Clough and Bent, 1998). Homozygous lines were selected in T3 generation and used for experiments.

### RNA Extraction and Q-PCR

The primer pairs to check for *PLC1* to *PLC9* expression were obtained from Tasma et al. (2008). Similarly, *CUC2-* and *MIR164A*-expression levels were determined with the primers described by Bilsborough et al. (2011). The primer pair to measure *PLC7* (At3g55940) expression levels was: 5′-GGCTTTCAATATGCAGGGACT-3′ and 5′-CGGGTCAAATAACAGCGTTGG-3′. Total RNA was extracted with Trizol reagent (Invitrogen, Carlsbad, CA). Total RNA (1.5 μg) from 10-day-old seedlings, or 4-week old rosette leaves, were converted to cDNA using oligo-dT18 primers, dNTPs and SuperScript III Reverse Transcriptase (Invitrogen), according to the manufacturer’s instructions. Q-PCR was performed with ABI 7500 Real-Time PCR System (Applied Biosystem). The relative gene expression was determined by comparative threshold cycle value. Transcript levels were normalized by the levels of *SAND* (At2g28390; forward primer: 5′-AACTCTATGCAGCATTTGATCCACT-3′, reverse primer: 5′-TGAAGGGACAAAGGTTGTGTATGTT-3′; Hong S.M. et al., 2010) or OTC (At1g75330; forward primer: 5′-TGAAGGGACAAAGGTTGTGTATGTT-3′, reverse primer: 5′-CGCAGACAAAGTGGAATGGA-3′) (Bilsborough et al., 2011; Han et al., 2013). Three biological- and two technical replicates were performed for means and standard deviations (Han et al., 2013).

### Histochemical GUS Analysis

GUS staining was performed as described previously (Zhang et al., 2018a,b). Briefly, transgenic plants carrying *pPLC7::GUS*-*SYFP* were grown for indicated times, after which specific tissues were taken and incubated in an X-Gluc reaction solution containing 1 mg/ml 5-bromo-4-chloro-3 indolyl-β-D-glucuronic acid (X-gluc), 50 mM phosphate buffer (pH 7.0), and 0.1% TX-100. Material was incubated overnight at 37°C and the next day cleared by 70 % ethanol and kept in that solution. GUS staining was visualized under a stereo microscope (Leica MZFLIII) and digitalized with a ThorLabs, CCD camera.

### Seed Staining and Sugar Analysis

To visualize seed coat mucilage, mature dry seeds were stained as described in Macquet et al. (2007). Seeds were directly incubated in 0.03% (w/v) Ruthenium red, or after imbibition in 0.5 M EDTA, pH 8.0, for 90 min. For the latter, seeds were washed with water to remove the EDTA and then stained for 20 min with Ruthenium red. Stained seeds were routinely observed with a light microscope (Aristoplan; Leitz). To visualize the surface and the adherent mucilage (AM) layer by confocal microscopy, Calcofluor white (0.01%) and Pontamine S4B were used as staining solutions (Western et al., 2000; Saez-Aguayo et al., 2014). Optical sections were obtained with an Olympus LX81 spectral confocal laser-scanning microscope. A 405 nm diode laser was used to excite Calcofluor white and the emission detected between 412 and 490 nm. For Pontamine S4B, a 561 nm diode laser was used and the detection performed between 570 and 650 nm. For comparisons of the signal intensity within one experiment, the laser gain values were fixed. Three different batches of seeds were analyzed and all of them showed the same phenotype. LUT green fire blue filter and LUT fire filter (Image J) were applied to the Calcofluor white and Pontamine S4B images, respectively.

Soluble carbohydrates were determined as described by Ribeiro et al. (2014), with minor modifications. Three milligrams of dry seeds were ground to powder afterwhich 1 mL of methanol (80% v/v) was added together with 40 μg mL-1 melezitose as internal standard. Samples were incubated in a water bath for 15 min at 76°C before being completely dried by vacuum centrifugation. After addition of 500 μL milliQ water, samples were thoroughly vortexed and centrifuged for 5 min at 17000 ×*g*. The supernatant was injected onto a Dionex HPLC system (Dionex, Sunnyvale, CA, United States) consisting of a gradient pump module (model GP40), a CarboPac PA100, 4 mm × 50 mm guard column, CarboPac PA100 4 mm × 250 mm seperating column, and an ED40-pulsed electrochemical detector. Soluble carbohydrates were separated by elution with increasing concentrations of NaOH (50–200 mM) at a flow rate of 1 mL min-1. Peaks were identified by co-elution of standards. Quantities were corrected via the internal standard and transformed into μg sugar per mg dry weight.

### Leaf-Shape Analysis

Rosettes from 4-week old plants, grown under long-day condition (22°C; 16 h light/8 h darkness), were detached and photographed immediately. Leaves were subsequently removed from the rosette, adhered to white paper using clear adhesive tape and then scanned (Epson Perfection V700 scanner). The 8^th^ leaf was used for calculation. Blade length, -width, -perimeter, -area, -serration number and serration levels were calculated from silhouettes using ImageJ software. Leaf-serration levels are expressed as the distance from tip-to-midvein divided by the distance from sinus-to-midvein, for indicated tooth (2nd–4th) (Kawamura et al., 2010).

### Stomatal Aperture

Stomatal aperture measurements were performed according to Distéfano et al. (2012) with minor modifications. Treatments were performed on epidermal strips excised from the abaxial side of fully expanded Arabidopsis leaves of 3-week-old plants, grown at 22°C under 16 h of light and 8 h of dark. Strips were immediately floated onto opening buffer (5 mM MES-KOH, pH 6.1, 50 mM KCl) for 3 h, and subsequently transferred to opening buffer ± ABA. After 90 min, stomata were digitized using a Nikon DS-Fi 1 camera, coupled to a Nikon Eclipse Ti microscope. Stomatal-aperture width was measured using ImageJ software (National Institute of Health).

### ^32^P_i_-Phospholipid Labeling, Extraction and Analysis

Developing seeds at 10 DAP were carefully removed from the silique. Mature seeds were sterilized and stratified on ½MS (pH 5.8) plates as described, and germinated under long-day conditions for around 20 h when testa ruptured. Both developing and germinating seeds were then transferred to 200 μl labeling buffer (2.5 mM MES, pH 5.8, 1 mM KCl) containing 5–10 μCi ^32^PO_4_^3-^ (^32^P_i_) (carrier free; Perklin-Elmer) in a 2 ml Eppendorf Safelock tube for 24 h.

Five-day-old seedlings were incubated O/N in labeling buffer and the next day labeled for 3 h. Samples were treated by adding 200 μl labeling buffer ± sorbitol (final concentration, 300 mM) for 30 mins. Labeling and treatments were stopped by adding perchloric acid (final concentration, 5% by vol.) for 5–10 min, after which lipids were extracted and phospholipids separated by TLC (Munnik and Zarza, 2013). Radioactive phospholipids were visualized by autoradiography and quantified by phosphoimaging (Molecular Dynamics, Sunnyvale, CA, United States). Individual phospholipid levels are expressed as the percentage of total ^32^P-lipids.

### Drought Tolerance Assays

Drought assays were performed as described (Zhang et al., 2018a,b). In brief, seeds were stratified at 4°C in the dark and sowed in pots (4.5 cm × 4.5 cm × 7.5 cm) containing equal amounts (80 g) of soil. Nine plants per pot were grown under short-day conditions (22°C with 12 h light/12 h dark) for 4 weeks and then subjected to dehydration by withholding them for water for 2 weeks while control plants were normally watered. Each experiment used 36 plants per genotype and experiments were repeated at least twice. To assay water-loss, rosettes from 4-week-old plants were detached and the FW determined every hour by weighing. Water content was calculated as the percentage from the initial FW. Twenty plants were used for each experiment and independently repeated twice.

## Results

### Expression of *PLC7* During Plant Development

**FIGURE 1:**
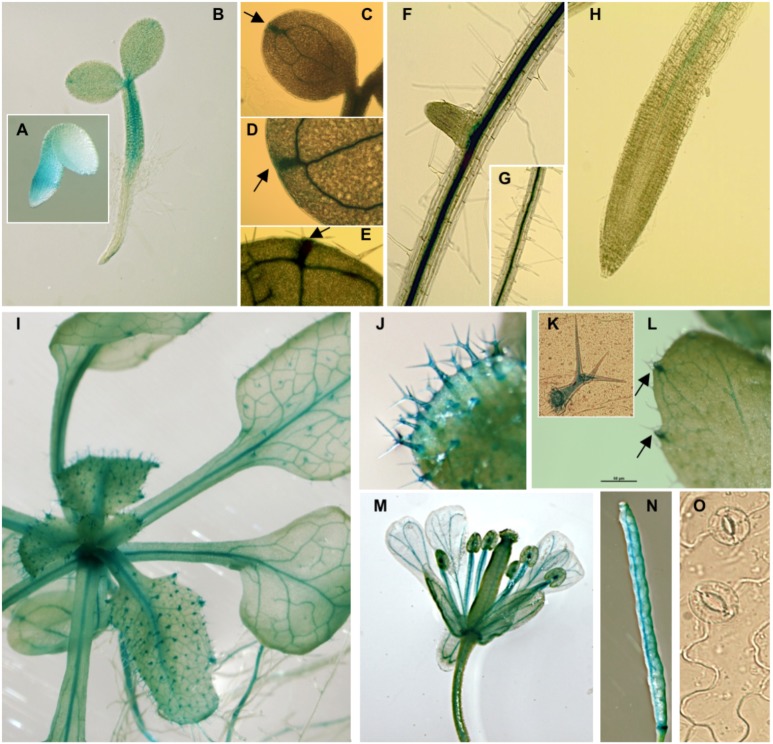
pPLC7-GUS expression in seedlings and specific tissues of Arabidopsis. **(A)** Embryo, 28 hrs after stratification, **(B)** vascular tissue of 2-d old seedlings, **(C–H)** 10-d old seedlings, including cotyledons and roots, **(I)** 3-week-old mature plant, **(J,K)** trichomes, **(C–E,I,L)** hydathodes (indicated by arrows), **(M)** flower, including style, filament, receptacle and pedicel, **(N)** silique, **(O)** guard cells (no staining detectable).

Histochemical GUS analyses on *pPLC7*-*GUS-SYFP* reporter lines indicated that *PLC7* was mainly expressed in the vasculature throughout all stages of development, including root, cotyledons, leaves, hypocotyl, flower (stamen, style, petal, sepal, receptacle and pedicel) and silique septum (Figure 1), which is similar to the pattern of *PLC3* (Zhang et al., 2018a) and *PLC5* (Zhang et al., 2018b). After germination, *PLC7* expression was mainly observed in the hypocotyl (Figures 1A,B), which then spread to the vasculature throughout the plant upon further development (Figures 1C–I). Interestingly, expression was quite abundant at hydathodes, both in young seedling and mature plants (Figures 1B–E,L, indicated by arrows). Unlike *PLC3* and *PLC5*, *PLC7* did not display the characteristic, “segmented” expression in the root vasculature. Instead, expression was homogenous in both main root- and lateral root vasculature (Figures 1F,G), and stopped near the transition zone (Figure 1H). GUS staining was also visible in trichomes (Figures 1J,K), similar to *PLC5* but stronger, and different from *PLC3*, which only showed expression at the base of the trichome (Zhang et al., 2018a,b). In contrast to *PLC3* and *PLC5*, no GUS activity was detected in guard cells (Figure 1O). We also tried to image the YFP signal but this was unfortunately too low.

While our results confirm the Q-PCR data from Tasma et al. (2008), that *PLC7* is expressed throughout the plant, our results indicated that this expression is primarily restricted to the vasculature, hydathodes and trichomes.

### *plc7* and *plc5/7* Mutants Show Wild Type Root Growth

**FIGURE 2:**
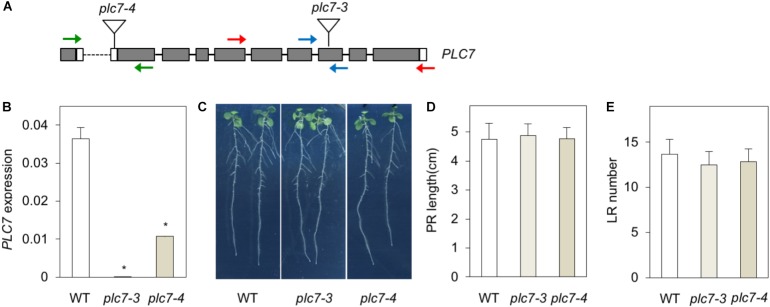
KO- or KD of *PLC7* does not affect root development. **(A)** T-DNA insertion positions (triangles) in the *PLC7* gene of the *plc7-3* (KO) and *plc7-4* (KD) lines. Filled boxes and lines represent exons and introns, respectively, while open boxes represent untranslated regions. Blue primers were used for qPCR analyses, red primers for genotyping *plc7-3*, and green primers for genotyping *plc7-4*. Forward primer *PLC7-4* is located at the last exon and 3’UTR of the gene in front. **(B)** *PLC7* expression levels in wild-type, *plc7-3* and *plc7-4* measured by Q-PCR using *SAND* as a reference gene. Values are the means ± SD (*n* = 3) of a representative experiment that was independently repeated three times. **(C)** Seedling morphology of wild type and *plc7* mutants. Seeds were germinated on ½MS with 0.5% sucrose for 4 days and then transferred to ½MS plates without sucrose. Photographs were taken 12 days after germination (DAG). **(D)** Primary root (PR) length and **(E)** lateral root (LR) number at 12 DAG. Values are means ± SE of three independent experiments (*n* > 20). ^∗^Indicates significance at *P* < 0.05 compared to wild-type, based on Student’s *t*-test.

To functionally address the role of *PLC7*, two homozygous T-DNA insertion lines, *plc7-3* (SALK_030333) and *plc7-4* (SALK_148821) were obtained (Figure 2A). *PLC7* expression was validated by Q-PCR and revealed that *plc7-3* is a KO- and *plc7-4* a KD mutant (Figure 2B). In contrast to *plc3-* (Zhang et al., 2018a) and *plc5* mutants (Zhang et al., 2018b), the root architecture of *plc7* mutants did not differ from wild type (Figures 2C,D).

To analyze gene redundancy, we tried to generate *plc3/5/7*-triple mutants by crossing *plc7-3* with the *plc3/5*-double mutant (Zhang et al., 2018b). After genotyping T2- and T3 populations, we could not identify any *plc3 plc7*-double mutants nor any homozygous triple mutants. We did find homozygous *plc5/7*-double mutants (Supplementary Figure S1) but as shown in Supplementary Figures S1B–D, no significant differences between the root systems of *plc5/7-* and wild-type seedlings were found.

### *plc5/7* Mutant Displays Mucilage Defect

**FIGURE 3:**
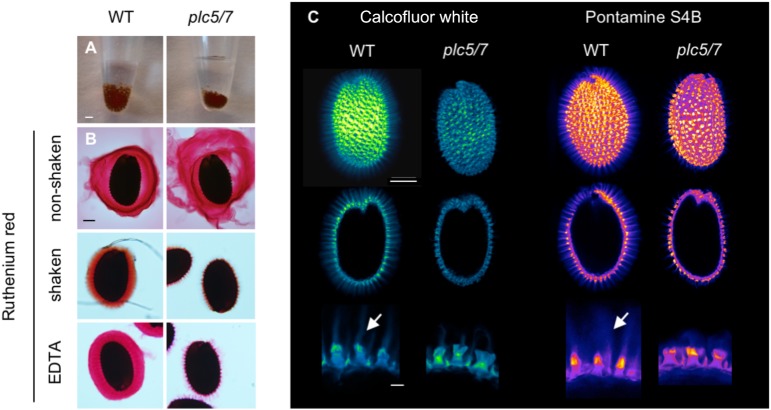
Seeds of *plc5/7*-double mutant exhibit a mucilage defect. **(A)** Seeds of *plc5/7* swell less than WT during imbibition. Equal amounts of dry seeds of WT and mutant were immersed in water O/N and photographed the next day. **(B)** Ruthenium red staining of wild type- and *plc5/7* seeds without shaking (*top*), with shaking (*middle*), or EDTA treatment (*bottom*). ’*With’* shaking shows adherent mucilage layer; ’*without’* shaking displays both adherent and non-adherent mucilage layers. **(C)** Cellulose staining by Calcofluor white (left panel) or Pontamine B (right panel) in wild type- and *plc5/7* seeds. Confocal images of whole seeds, cross section, and close-up views (top, middle and bottom, respectively) are shown. Bars represent 2 mm **(A)**, or 0.1 mm (*top* and *middle* rows) or 0.025 mm (*bottom* row) **(B)**. Representative results of at least 3 biological replicates are shown.

While imbibing seeds for stratification, we noticed that the volume of the *plc5/7*-seed pellet was always smaller than wild-type’s after O/N incubation (Figure 3A). This was not the case for the individual *plc7* or *plc5* mutants (Supplementary Figure S2; Zhang et al., 2018b). Upon imbibition, the seed coat-epidermal cells normally extrude mucilage that forms two transparent layers (adherent and non-adherent layers) around the seed. To examine whether the smaller volume of the *plc5/7* mutant was caused by a mucilage defect, we stained imbibed seeds with Ruthenium red (Figure 3B) that stains pectins, the main component of mucilage (Golz et al., 2018). Compared to wild-type, the adherent and non-adherent layers were more expanded in the *plc5/7* mutant (Figure 3B, top panel) and when seeds were mildly shaken to remove the non-adherent layer, or treated with EDTA, *plc5/7* seeds lost their adherent layer completely (Figure 3B, middle and lower panel, respectively).

Increased solubility of the pectins has been linked to perturbation of cellulose deposition (Basu et al., 2016; Ben-Tov et al., 2018). To test this, wild type- and *plc5/7* seeds were stained with Calcofluor White (CFW, for cellulose and other β-glucans staining; (Figure 3C, left panel) or Pontamine S4B (cellulose-specific dye; Figure 3C, right panel) (Anderson et al., 2010; Wallace and Anderson, 2012). In wild-type seeds, the primary cell wall remnants and rays extending from the columella were stained by both dyes (Figure 3C). The staining pattern of *plc5/7* seeds appeared similar, but the CFW intensity was lower and the rays were clearly reduced compared to wild-type. These results point to a role for PLC5 and PLC7 in cellulose-ray formation, which is a novel function for PLCs.

### *PLC5* and *PLC7* Are Expressed in Developing Seeds

**FIGURE 4:**
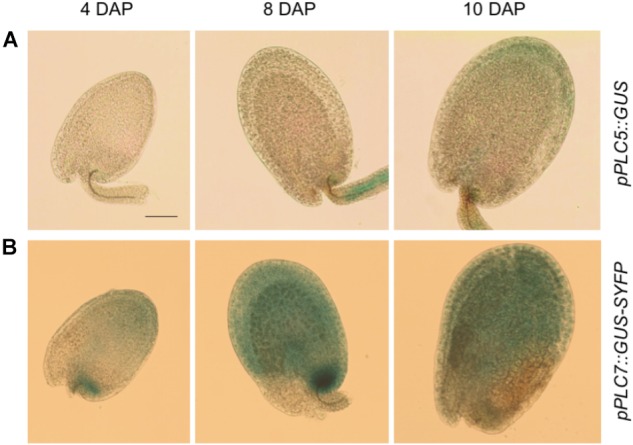
*PLC5* and *PLC7* expression during seed development. **(A)** GUS activity analysis in *pPLC5::GUS-*developing seeds. Expression was found in the funiculus at 8 days after pollination (DAP) and in the seed coat at 10 DAP. **(B)** GUS activity analysis in *pPLC7::GUS-SYFP*-developing seeds. Staining was found in the chalazal and seed coat. Representative results of 3 independent experiments are shown. Bar = 0.1 mm.

To investigate the expression of *PLC*5 and *PLC7* during seed development, additional histochemical GUS analyzes were performed (Figure 4). At 4 days after pollination (DAP), some GUS activity was found for *PLC5* (Figure 4A) while a strong staining in the seed coat and chalazal area for *PLC7* was obtained (Figure 4B). Later in development (8 and 10 DAP), expression increased, with *PLC5* expression appearing in the seed coat and funiculus (Figure 4A), and *PLC7* becoming stronger in the seed coat and chalazal (Figure 4B).

### PPI- and PA Levels in Developing and Germinating Seeds

**FIGURE 5:**
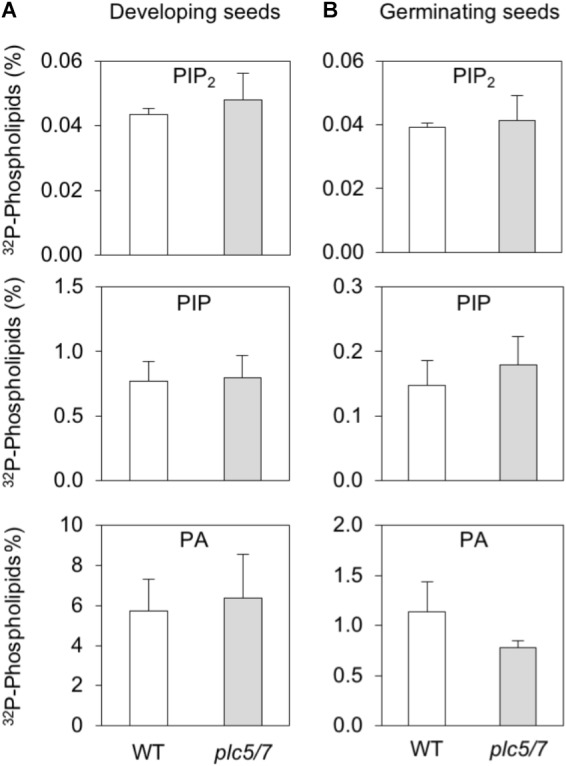
PPI- and PA levels in developing- and germinating (mature) seeds of wild type and *plc5/7*. **(A)** Developing seeds (∼00; 10 DAP) or **(B)** pre-germinated mature seeds (∼200) of wild type and *plc5/7* were labeled with ^32^P_i_ for 24 h and their lipids extracted, separated by TLC and quantified by Phosphoimaging.^32^P-levels of PIP_2_, PIP and PA are expressed as percentage of total ^32^P-phospholipids. Three independent experiments were performed; data shown are means ± SD (*n* = 3) of a representative experiment.

To analyze substrate- (i.e., PIP and PIP_2_) and product- (conversion of PLC-generated DAG into PA) relationships, wild type- and *plc5/7* seeds at 10 DAP were compared with germinating, mature seeds after 24 h of ^32^P_i_-labeling. As shown in Figure 5, wild-type and *plc5/7* seeds contained similar amounts of PIP_2_, PIP and PA in both stages. Interestingly, PIP- and PA levels were much higher in developing seeds, while PIP_2_ levels were similar in both stages.

### Enhanced Leaf Serration Phenotype for *plc5/7* Plants

**FIGURE 6:**
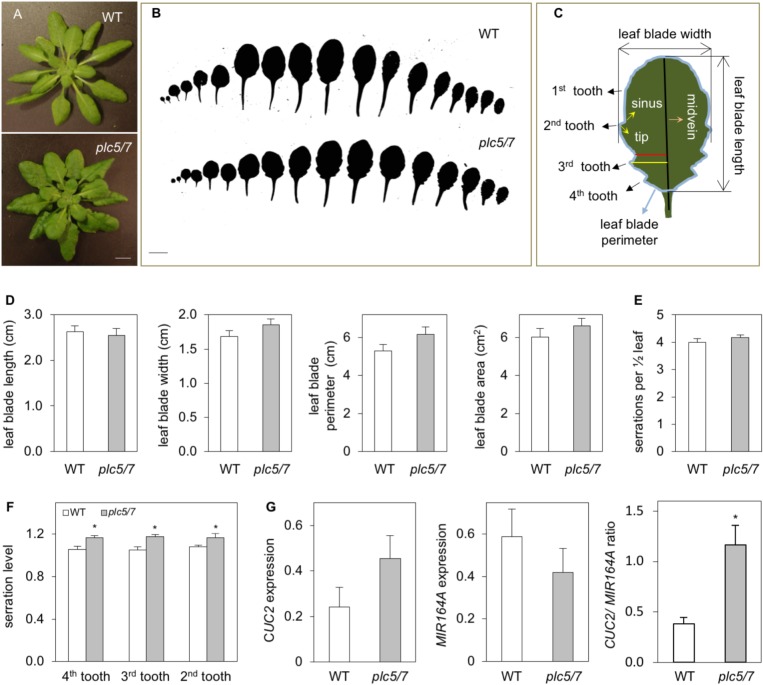
Leaves of *plc5/7* plants display enhanced leaf serration. **(A)** Rosette of wild type and *plc5/7* mutant. Plants were grown on soil for 4 weeks in short-day conditions, afterwhich rozettes were cut and photographed immediately. Bar = 1 cm. **(B)** Leaf series of 4-week old wild type and *plc5/7* plant. **(C)** Cartoon demonstrating the leaf parameters measured of the 8^th^ leaf. **(D)** Quantification of blade size including, length, width, perimeter and area. **(E,F)** Quantification of leaf serration number **(E)** and level **(F)** in wild type and *plc5/7* mutant. **(G)** Expression of *CUC2* and *MIR164A* and their ratio in WT and *plc5/7* mutant, relative to the expression of reference gene, *OTC*. Data represents the means ± SD (*n* = 3) from a representative experiment that was repeated twice with similar results. Asterisk (^∗^) indicate significance at *P* < 0.05 compared to WT, based on Student’s *t*-test.

Growing *plc5/7* mutants on soil revealed a novel phenotype, i.e., the patterning of their leaf-edge (serration). This phenotype was absent from the individual *plc7* or *plc5* mutants (Supplementary Figure S3; Zhang et al., 2018b). Overall, the level of serration in successive rosette leaves was significantly increased in *plc5/7*, which appeared to be stronger in the proximal part of the blade than in the distal part (Figures 6A,B). To quantify this in more detail, we measured various parameters of the 8th leaf (Figures 6C–E) of 4-weeks old rosettes of both genotypes. No changes in blade length were observed between wild type and *plc5/7*. Blade width, -perimeter and -area, appeared to be slightly bigger in *plc5/7* but this was not significant (Figure 6D). The serration number neither changed, but the serration level (indicated by the ratio between the distance from the midvein to tip and the distance from the midvein to sinus; Figure 6C) was significantly higher in three successive teeth (Figure 6F).

In Arabidopsis, leaf-margin development is controlled by a balance between *microRNA164A* (*MIR164A*) and *CUP-SHAPED COTYLEDON2 (CUC2)* (Nikovics et al., 2006). Hence, we compared the expression of *MIR164A* and *CUC2* in wild type and *plc5/7* leaves. As shown in Figure 6G, *plc5/7* leaves were consistently found (three independent experiments) to contain higher levels of *CUC2* and lower levels of *MIR164A*, resulting in a significant increase in the *CUC2*/*MIR164A* ratio.

### *plc5/7* Mutants Are Better Protected Against Drought

**FIGURE 7:**
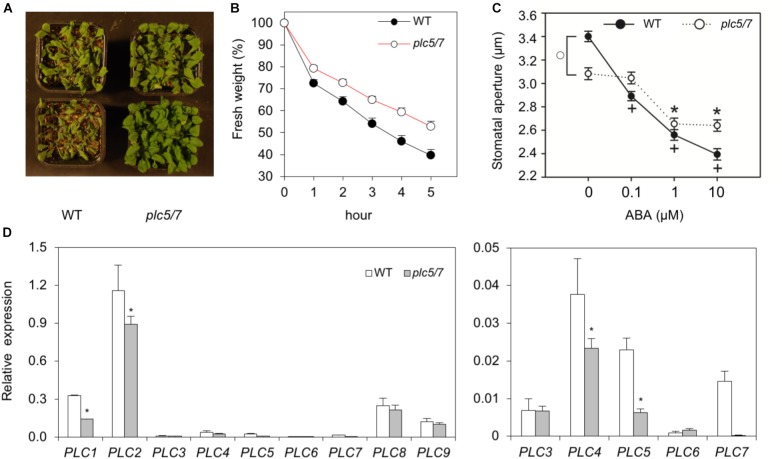
Soil grown-*plc5/7* plants are more tolerant to drought stress. **(A)** Six-weeks old plants of wild type- or *plc5/7*, grown on soil and exposed to drought by withholding water for the last 2 weeks. **(B)** Water loss of detached rosettes of normally watered, 4-weeks old plants. Water loss was measured at indicated time points and expressed as a percentage of the initial fresh weight. Values are means ± SD for one representative experiment for 3 independent experiments (*n* = 36). **(C)** Effect of ABA on the stomatal aperture in leaf strips of wild type and *plc5/7* plants. Values are means ± SE of at least three independent experiments (*n* > 100). **(D)** Left panel: *PLC1- PLC9* expression levels in wild type- and *plc5/7* leaves measured by Q-PCR, relative to *SAND* expression. Right panel: Zoom-in of the expression of *PLC3-PLC7*. Values are means ± SD (*n* = 3) for a representative experiment that was repeated twice with similar results.

When plants were left in the greenhouse without watering, we noticed that *plc5/7* mutants appeared to be more drought tolerant while single mutants behaved like wild type (data not shown). Performing multiple drought assays confirmed this (Figure 7A), while detached rosettes of 4-week-old *plc5/7* plants lost less water than wild type (Figure 7B).

ABA plays a key role during the response to dehydration stress and is known to induce stomatal closure to reduce water loss (Sean et al., 2010). Hence, we checked the stomatal-closure of *plc5/7* and wild type in response to ABA. As shown in Figure 7C, *plc5/7* has less-open stomata compared to wild type without ABA, while upon ABA treatment, the *plc5/7* mutants were less responsive.

Previously, we showed that overexpression of *PLC3* or *PLC5* enhanced drought tolerance in Arabidopsis (Zhang et al., 2018a,b), which was found earlier for maize, canola and tobacco (Wang et al., 2008; Georges et al., 2009; Tripathy et al., 2011). We therefore wondered whether the increase in drought tolerance in *plc5/7* was a result of the overexpression of any of the other (redundant) *PLCs*. However, no strong overexpression of any *PLC* was found in *plc5/7* (Figure 7D). In fact, *PLC1*, *PLC2* and *PLC4* appeared to be slightly down-regulated (Figure 7D).

### Overexpression of *PLC7* Increases Drought Tolerance

**FIGURE 8:**
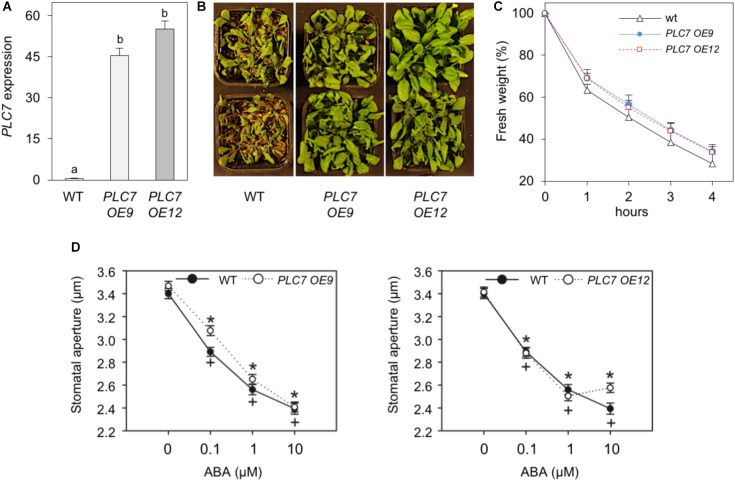
Overexpression of *PLC7* enhances drought tolerance. **(A)** *PLC7* expression levels in wild type and two homozygous *PLC7*-overexpression lines, *PLC7-OE9* and *PLC7-OE12* as measured by Q-PCR relative to *SAND*. Values are means ± SD (*n* = 3) for one representative experiment. **(B)** Phenotype of 6-week old plants from wild type and *PLC7-*overexpresion lines, *#OE9* and *#OE12*, after 2 weeks of drought. **(C)** Water loss of detached rosettes of 4-weeks old plants. Values are means ± SD for one representative experiment for 3 independent experiments (*n* = 36). **(D)** Stomatal aperture in leaf peels of wild type, *PLC7-OE9* (*left*), *PLC7-OE12* (*right*) and the effect of ABA. Values are means ± SE of at least three independents (*n* > 100).

As mentioned above, overexpression of Arabidopsis *PLC3* or *PLC5*, which are from different subfamilies than *PLC7*, resulted in enhanced drought tolerance (Zhang et al., 2018a,b). To check the effect of the overexpression of *PLC7*, homozygous T3 plants were generated. Two lines, *PLC7-OE9* and *PLC7*-*OE12*, which overexpressed *PLC7* around 80- to 100-fold, respectively, were selected for further studies (Figure 8A). Both lines were found to be more drought tolerant than WT (Figure 8B), and lost slightly less water when rosettes of 4-week-old plants were detached (Figure 8C). The stomatal aperture and their response to ABA was found to be similar to wild type (Figure 8D), which is different from *PLC3-* and *PLC5-OE* plants that had more closed stomata than wild type.

### Phospholipid Responses in Osmotically Stressed Seedlings

**FIGURE 9:**
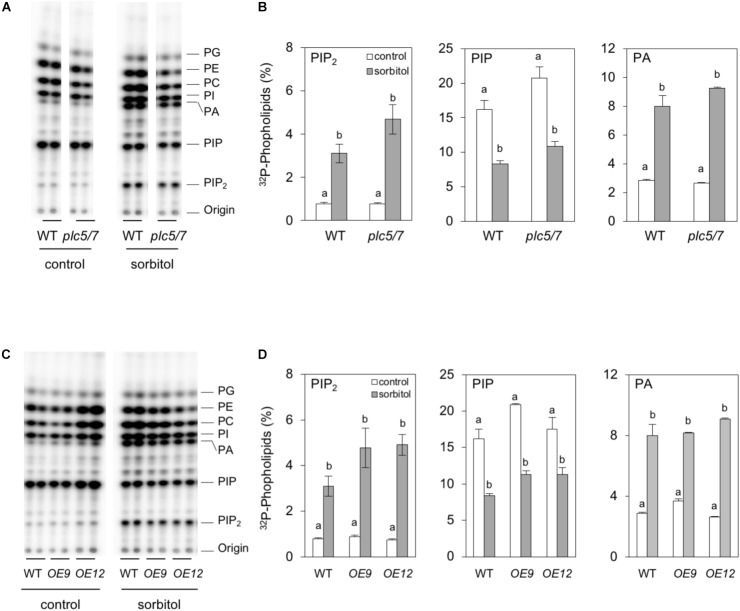
Hyperosmotic stress triggers stronger PIP_2_ responses in *plc5/7*- and *PLC7*-OE seedlings than WT. Six-day-old seedlings were ^32^P-labeled for 3 h and then treated with or without 300 mM sorbitol for 30 min. Extracted lipids were analyzed by TLC and quantified through phosphoimaging. **(A,B)** WT vs *plc5/7* seedlings, **(C,D)** WT vs *PLC7-OE* lines *#*9 and #12. **(A,C)** Typical TLC profiles, **(B,D)** ^32^P-levels in PIP_2_, PIP and PA. Data shown are the means ± SE (*n* = 3) of one experiment, representative of three independent experiments. Data was analyzed by 2-way ANOVA. Statistical significant differences between normal and sorbitol conditions in wild type and *plc5/7* or *PLC7-OEs* are indicated by letters (*P* < 0.05).

To analyze phospholipid responses in *plc5/7* and the *PLC7-OE* lines, ^32^P-labeling experiments (3 h pre-labeling) were performed on seedlings and the effect of sorbitol tested to mimic osmotic stress. Both *plc5/7* and wild type showed similar PPI- and PA levels in the absence of sorbitol (Figures 9A,B). Upon sorbitol treatment, a consistent stronger PIP_2_ response was observed for *plc5/7* seedlings in all three independent experiments (*P*-value almost 0.05). While PIP_2_ levels increased by ∼4 times in wild type, in *plc5/7* seedlings a typical 6-times increase was found. No such differences in PA- or PIP were observed (Figure 9B).

*PLC7-OE* lines revealed no difference in PPI- or PA levels compared to wild-type under control conditions (Figures 9C,D). However with sorbitol, again a stronger PIP_2_ response was observed in *PLC7-OE* lines, i.e., ∼6-times vs. ∼4-times increase for WT. PA- and PIP responses were similar to wild type (Figures 9C,D). These results suggest that both *plc5/7* and *PLC7*-*OE* plants boost more PIP_2_ in response to osmotic stress than wild-type, similar to what we found earlier for *PLC3-* and *PLC5-OE* lines (Zhang et al., 2018a,b).

## Discussion

### Knockout of *PLC7* Does Not Affect Root Architecture

Previously, we demonstrated that Arabidopsis *PLC3* and *PLC5* were both involved in lateral root formation, but that the phenotype in a *plc3/5*-double mutant was not worse than the individual, single mutants. Hence, we speculated that another PLC might be involved (Zhang et al., 2018a,b). Since promotor-GUS analyses of *PLC3* and *PLC5* revealed specific expression in phloem companion cells and showed a typical ‘segmented’ pattern in the vascular of the primary root from which lateral roots emerged, we searched for other phloem-specific PLCs. Using the eFP browser, we found two potential candidates, *PLC2* and *PLC7*. Since T-DNA insertion mutants of *PLC2* were lethal (Li et al., 2015; D’Ambrosio et al., 2017; Di Fino et al., 2017), we focused on *PLC7*. Two independent homozygous T-DNA insertion mutants were obtained, with *plc7-3* being a KO- and *plc7-4* a KD line. Both mutants, however, exhibited normal root architecture (Figure 2). In an attempt to create double- and triple mutants of the potentially redundant *PLCs*, we crossed *plc3-2/plc5-1* (*plc3/5*; Zhang et al., 2018b) with *plc7-3*, however, this only resulted in viable *plc5/7* double mutants, as the combination *plc3/plc7* turned out to be lethal (not shown). The *plc5/7* double mutant, however, did not reveal significant changes in root morphology (Figure 2 and Supplementary Figure S1). While *pPLC7-GUS* analyses confirmed the vascular expression of *PLC7*, which according to the Arabidopsis eFP Browser (Winter et al., 2007) is all phloem and phloem companion cells, it lacked the typical segmented pattern as found for *PLC3* and *PLC5* (Figure 1; Zhang et al., 2018a,b). These results may indicate that PLC2 and PLC3 represent redundant PLCs in root development. Interestingly, *PLC7* was also expressed in hydathodes and in seeds, which correlates well with the two new phenotypes that were found for *plc5/7*-double mutants, and have never been observed before. These include a mucilage phenotype in seeds and a serration phenotype in leaves. The latter may correlate with *PLC7’*s specific expression at the hydathodes. *PLC7* was also strongly expressed in trichomes. *PLC5* is also expressed in trichomes, although less, and *PLC3* is typically expressed at the basal cells of trichomes in developing leaves (Zhang et al., 2018a,b). We checked for trichome phenotypes in individual *plc3, plc5 or plc7* and *plc3/5-* and *plc5/7-* mutants, but found no obvious differences (number, shape). Again, this could be due to redundancy. Without defects, the role of PLC in trichomes remains unclear.

Another new finding for PLC loss-of-function mutants is that *plc5/7* mutants were more drought tolerant, a phenotype that is typically found when *PLC* is overexpressed (Wang et al., 2008; Georges et al., 2009; Tripathy et al., 2011; Zhang et al., 2018a,b). No upregulation of redundant PLCs in the *plc5/7* background was found so it must be a consequence of the *plc5/7* combination, possible in combination with the slight down regulation of *PLC1*, *PLC2* and *PLC4* that was found. RNASeq analyses of the *plc* mutant- and OE lines may shed light on potential pathways that are up- or down-regulated to explain various phenotypes. Most importantly, altering expression of *PLC* genes results in clear defects, which will help elucidating the roles PLC can play in plant signaling and development.

While new phenotypes provide new pieces of the PLC-signaling puzzle, it remains unclear how this is achieved at the cellular and molecular level. Flowering plants lack the prime targets for IP_3_ and DAG, but there are indications that plant responses are coupled via inositol polyphosphates (IPPs) and/or PA (Munnik, 2001; Arisz et al., 2009, 2013; Munnik and Zarza, 2013; Testerink and Munnik, 2011; Gillaspy, 2013; Munnik, 2014; Laha et al., 2015; Heilmann, 2016a,b; Hou et al., 2016; Noack and Jaillais, 2017; Yao and Xue, 2018). Alternatively, since PIP or PIP_2_ are emerging as second messengers themselves, PLC could function as an attenuator of signaling (Gillaspy, 2013; Munnik, 2014). In that respect, it is interesting to notice that the increased drought-tolerant phenotype in *plc5/7* and *PLC3*, *-5*, and *-7* overexpression lines correlates well with stronger PIP_2_ responses upon osmotic stress. How this works remains unclear, however. Maybe they are more primed to enhanced PIP_2_ turnover.

### Role for *PLC5* and *PLC7* in Seed Mucilage

The mucilage extrudes from seed coat epidermal cells when seeds are exposed to water, which helps seeds to remain hydrated while the germination process is in progress (Western et al., 2000). The major component of mucilage is pectin, of which polygalacturonic acid (PGA) and rhamnogalacturonan I (RGI) are the most common compounds (Carpita and Gibeaut, 1993; Cosgrove, 1997). In addition, several other polysaccharides can be found (containing arabinose, galactose, glucose, xylose and mannose), and are equally important in determining mucilage’s properties (Voiniciuc et al., 2015a,b). Two layers of mucilage can be distinguished, a water- soluble non-adherent outer layer and an adherent inner layer (Zhao et al., 2017). While the non-adherent outer layer is easily removed from the seed, the latter is relatively hard to detach, even chemically (Zhao et al., 2017). The *plc5 plc7* double mutant releases mucilage that is less adherent to seeds than in the wild type (Figure 3). In addition, cellulosic rays stained with calcofluor white and pontamine S4B appear shorter around the mutant seeds. These defects have been reported in mutants that disrupt cellulose synthesis (Mendu et al., 2011; Sullivan et al., 2011; Griffiths et al., 2015; Griffiths and North, 2017; Ben-Tov et al., 2018), as well as in mutants that directly impair the synthesis of xylan (Voiniciuc et al., 2015a,b; Hu, 2016; Hu et al., 2016; Ralet et al., 2016). Therefore, PLC could influence membrane phospholipids that are important for the trafficking of cellulose synthase enzymes or the secretion of matrix polysaccharides (such as xylan) from the Golgi apparatus. We checked for sugar-composition aberrations in whole seeds, but could not find changes compared to wild type (Supplementary Figure S4). Possibly, the distribution between non-adherent and adherent mucilage layers might be different, which needs to be further determined.

Histochemical analysis of the GUS-reporter lines indicated that both *PLC5* and *PLC7* are expressed during seed development (Figure 4). Until now, no mucilage deficiency has been linked to *PLC*, and we only observed the mucilage defect in the *plc5/7*-double mutant of all our *PLC* knockout mutants. The defect in both *PLC5* and *PLC7* probably breaks the balance for mucilage maintenance by altering cellulose deposition and/or crystallization in the inner mucilage. How the enzyme PLC could be involved in all this will require additional research. Potentially, PLC could be required for mucilage secretion or for the localization or activity of cellulose synthases, for example. PA, PIP and PIP_2_ have been implicated to play essential roles in vesicular trafficking, -fusion and -fission. Even though no difference was found in PPI- or PA levels in either developing or mature seeds (Figure 5), reduced amounts of PLC5 and lack of PLC7 might cause crucial local changes in lipid or IPP molecules.

### Role for PLC in Leaf Serration

Leaf shape is defined by the pattern- and degree of indentation at the margin area, distinguishing many plant species (Tsukaya, 2006). The patterning involves a complex cross-talk between hormone signaling and genetic regulators (Byrne, 2005; Fleming, 2005). The development of leaf serration involves auxin maxima at the protrusion of each serrated section (Hay and Tsiantis, 2006). Genetic studies identified the auxin efflux carrier PIN-FORMED 1 (PIN1) and CUP-SHAPED COTYLEDON 2 (CUC2) as two key factors required (Hay et al., 2006; Nikovics et al., 2006). PIN1 asymmetrically localizes on plasma membranes and directionally transports auxin, creating auxin maxima that direct the outgrowth of the serrations (Hay et al., 2006; Scarpella et al., 2006). CUC2 is a transcription factor that is post-transcriptionally downregulated in leaves by *MIR164A* (Nikovics et al., 2006). CUC2 expression is limited to the sinus where the serration starts, and the promotion of serration outgrowth is through cell division, not by suppression of sinus growth (Kawamura et al., 2010). CUC2 is also thought to regulate the polarized localization of PIN1 in convergence points at the leaf margin, where it may play a role in establishing, maintaining and/or enhancing auxin maxima that result in leaf serration (Bilsborough et al., 2011; Kawamura et al., 2010). In a feedback loop, auxin downregulates *CUC2*, both transcriptionally and post-transcriptionally through activation of *MIR164A* (Bilsborough et al., 2011).

The *plc5/7*-double mutant revealed a mildly-enhanced leaf-serration phenotype (Figure 6). Subsequent measurement of *CUC2-* and *MIR164A* expression revealed an up-regulation of *CUC2* and down-regulation *MIR164A*, consistent with enhanced serration ( Kawamura et al., 2010; Bilsborough et al., 2011). Promotor-GUS analyses showed that both *PLC5* and *PLC7* were expressed at leaf hydathodes, a secretory tissue that secretes water through the leaf margin that is associated with leaf serration (Tsukaya and Uchimiya, 1997) and auxin response maxima (Scarpella et al., 2006). Hence, it is possible that *PLC5* and *PLC7* redundantly contribute to the regulation of leaf serration, since the phenotype was absent in the single mutants. We also measured PPI-and PA levels in *plc5/7* rosette leaves, but like seeds and seedlings, we found no significant changes (data not shown; Figures 5, 9). It is possible that only small changes occur in particular cells and tissues but that most have normal levels/responses so that differences are lost in the total background. How *PLC5* and *PLC7* are involved in leaf serration requires further investigation.

### Role for PLC in Drought Tolerance

Earlier, overexpression of *PLC* in maize, tobacco and canola have been shown to improve their drought tolerance (Wang et al., 2008; Georges et al., 2009; Tripathy et al., 2011). Similarly, overexpression of *PLC3* or *PLC5* in Arabidopsis was shown to improve their drought tolerance (Zhang et al., 2018a,b), and here we show this also holds for *PLC7*. Interestingly, we also found an increased drought-tolerant phenotype for *plc5/7*-double mutants, which did not occur in the single *plc5-1* and *plc7-3* mutants. Previous results showed that *PLC3-OE* and *PLC5-OE* lines showed a reduced closing response to ABA compared to wild type and had less stomata open in the absence of ABA (Zhang et al., 2018a,b). Under control conditions, overexpression of *PLC7* did not reveal this “less-open stomata” phenotype, and their stomatal response to ABA was similar to wild type. Stomata of *plc5/7* plants were less open, which is in contrast to the *plc5-* and *plc7-*single mutants that had a normal opening at control conditions (Figure 7C and Supplementary Figure S5; Zhang et al., 2018b). However, *plc7* and *plc5/7* mutants were both less sensitive to ABA-induced stomatal closure (Figure 7C and Supplementary Figure S5). Under control conditions, *PLC7* is not expressed in guard cells (Figure 1) but levels are strongly upregulated upon ABA treatment (Bauer et al., 2013). Hence, the drought tolerant phenotype in *plc5/7* could be a consequence of a local upregulation (e.g., guard cells) of one or more redundant *PLCs*, which may remain undetectable when whole seedling-mRNA levels are measured (Figure 7D).

Salt- and hyperosmotic stress have been shown to trigger phospholipid-signaling responses in many studies (Hong Y. et al., 2010; Munnik and Zarza, 2013; Hou et al., 2016; Meijer et al., 2017). To mimic the osmotic stress of drought, the effect of sorbitol on ^32^P-prelabeled seedlings of *plc5/7*, *PLC7-OE* and wild type was measured. Under control conditions, no difference in PPIs- and PA levels were found among genotypes, however, in response to sorbitol, a much stronger PIP_2_ response in both *plc5/7* and *PLC7*-*OE* lines was found (Figure 9). Whether the above response in *plc5/7* is a consequence of enhanced, local expression of another *PLC*, or a consequence of differential gene expression and hence drought tolerant response, requires further studies. Overexpression of *PLC3* and *PLC5* also showed an enhanced PIP_2_ response. Maybe the constitutive hydrolysis of PPIs due to overexpression of PLC, enhances the activity of the lipid kinases to replenish PPI pools, and that hyperosmotic stress activation (sorbitol/drought) causes stronger PIP_2_ responses and, hence, downstream signaling. Apart from being a precursor of IPPs and PA, PIP_2_ is also emerging as signaling molecule itself, e.g., involving reorganization of the cytoskeleton, endo- and exocytosis, and ion channel regulation (Xue et al., 2009; Ischebeck et al., 2010; Heilmann and Heilmann, 2015; Heilmann, 2016a,b; Heilmann and Ischebeck, 2016; Gerth et al., 2017; Noack and Jaillais, 2017). Whether PLC performs as a signal generator or PIP_2_-signaling attenuator remains to be shown and investigated. The identification and characterization of some genuine PIP_2_ targets will be essential to start unraveling the molecular mechanisms involved.

## Author Contributions

RvW, QZ, XZ, ML, AL, and TM designed the experiments. ML, XZ, FM, and WL performed the mucilage experiments, while AG, DS, and CG-M performed the stomatal measurements. RvW, QZ, and ML executed all the remaining experiments. RvW, QZ, AL, MH, and TM wrote the article.

## Conflict of Interest Statement

The authors declare that the research was conducted in the absence of any commercial or financial relationships that could be construed as a potential conflict of interest.

## Footnotes

1 signal.salk.edu

2 www.lifetechnologies.com

## Supplementary Material

Figure S1 A *plc5/plc7* double mutant is viable and not affected in root architecture. **(A)** Q-PCR expression levels of *PLC5* (*left*) and *PLC7* (*right*) in wild type and *plc5/7*-double mutant, using *SAND* as reference gene. Values are means ± SD (*n* = 3) of a representative experiment that was repeated three times. **(B)** Seedling morphology of wild type and *plc5/7* (12 DAG). **(C)** Primary root (PR) length and **(D)** lateral root (LR) number at 12 DAG. Values are means ± SE of three independent experiments (*n* > 20). Asterisk (^∗^) indicate significance at *P* < 0.05 compared to wild-type, based on Student’s *t*-test.

Figure S2 Single *plc* mutants do no show defect in mucilage swelling. Equal amounts of dry seeds (∼1000) of WT, single plc5- and plc7-mutants, and *plc5/7* double mutants were immersed in water and photographed after 0 (upper panel) and 12 hrs imbibition (lower panel). Only the *plc5/7* double mutants exhibit a mucilage defect and therefor swell less than WT or the individual *plc* mutants.

Figure S3 Single *plc* single mutants do not show serration phenotype. Plants were grown for 4 weeks at short-day conditions and photographed. Two separate experiments are shown to show that single mutants look like WT and do not have the serration phenotype as observed for *plc5/plc7-*double mutants. **(A)** Plant were grown with either 9 plants/pot (top 3 rows) or 5 plants/pot (lower row). **(B)** Shows WT, single- and *plc5/plc7-*double mutants, with the latter clearly showing increased leaf serration.

Figure S4 Sugar composition of wild type- and *plc5/7-*mutant seeds. Sugars were extracted from dry seeds and quantified by HPAEC-PAD. Quantities were corrected through internal standards, and transformed into mg of sugar per gram of dry material. Values represent the means of triplicates ± SE of three independent experiments.

Figure S5 *PLC7*-KO and -KD mutants are less sensitive to ABA induced-stomatal closure. Leaves from 3-weeks old plants were stripped and peels were incubated in opening buffer with light for 3 h until stomata were fully open. Peels were then treated with different concentrations of ABA for 90 mins, after which stomata were digitized and the aperture width measured for wild type and *plc7-3* (*left*) or *plc7-4* (*right*). Data were analyzed by one-way ANOVA. For each plant line, treatments were compared against their own control and statistically significant differences between doses are indicated by crosses for wild type or asterisks for transgenic lines (*P* < 0.05, Dunn’s method). Wild type and different genotypes were compared in control conditions and statically differences are indicated with empty circle (*P* < 0.001, Mann Whitney). Values are represented by means ± SE of at least three independent experiments (*n* > 100).
