# Supplementary material for: Role for Arabidopsis PLC7 in Stomatal Movement, Seed Mucilage Attachment, and Leaf Serration
Source: Front Plant Sci. 2018 Nov 27;9:1721. doi: 10.3389/fpls.2018.01721 (PMC6278229; doi:10.3389/fpls.2018.01721)

SUPPLEMENTARY MATERIAL

Supplementary Figure S1| A *plc5/plc7* double mutant is viable and not affected in root architecture. (A) Q-PCR expression levels of *PLC5* (*left*) and *PLC7* (*right*) in wild type and *plc5/7*-double mutant, using *SAND* as reference gene. Values are means ± SD (n = 3) of a representative experiment that was repeated three times. **(B)** Seedling morphology of wild type and *plc5/7* (12 DAG). **(C)** Primary root (PR) length and **(D)** lateral root (LR) number at 12 DAG. Values are means ± SE of three independent experiments (n>20). Asterisk (*) indicate significance at P<0.05 compared to wild-type, based on Student's *t* test.

Supplementary Figure S2| Single *plc* mutants do no show defect in mucilage swelling.
Equal amounts of dry seeds (~1000) of WT, single plc5- and plc7-mutants, and *plc5/7* double mutants were immersed in water and photographed after 0 (upper panel) and 12 hrs imbibition (lower panel). Only the *plc5/7* double mutants exhibit a mucilage defect and therefor swell less than WT or the individual *plc* mutants.

Supplementary Figure S3| Single *plc* mutants do not show serration phenotype.
Plants were grown for 4 weeks at short-day conditions and photographed. Two separate experiments are shown to show that single mutants look like WT and do not have the serration phenotype as observed for *plc5/plc7*-double mutants. A) Plants were grown with either 9 plants/pot (top 3 rows) or 5 plants/pot (lower row). B) Shows WT, single- and *plc5/plc7*-double mutants, with the latter clearly showing increased leaf serration.

Supplementary Figure S4 | Sugar composition of wild type- and *plc5/7*-mutant seeds. Sugars were extracted from dry seeds and quantified by HPAEC-PAD. Quantities were corrected through internal standards, and transformed into mg of sugar per gram of dry material. Values represent the means of triplicates ± SE of three independent experiments.

Supplementary Figure S5 | *PLC7*-KO and -KD mutants are less sensitive to ABA induced-stomatal closure.
Leaves from 3-weeks old plants were stripped and peels were incubated in opening buffer with light for 3 h until stomata were fully open. Peels were then treated with different concentrations of ABA for 90 mins, after which stomata were digitized and the aperture width measured for wild type and *plc7-3* (*left*) or *plc7-4* (*right*). Data were analyzed by one-way ANOVA. For each plant line, treatments were compared against their own control and statistically significant differences between doses are indicated by crosses for wild type or asterisks for transgenic lines (P < 0.05, Dunn's method). Wild type and different genotypes were compared in control conditions and statically differences are indicated with empty circle (P <0.001, Mann Whitney). Values are represented by means ± SE of at least three independent experiments (n >100).

Supplemental figure S1 – Van Wijk *et al.*

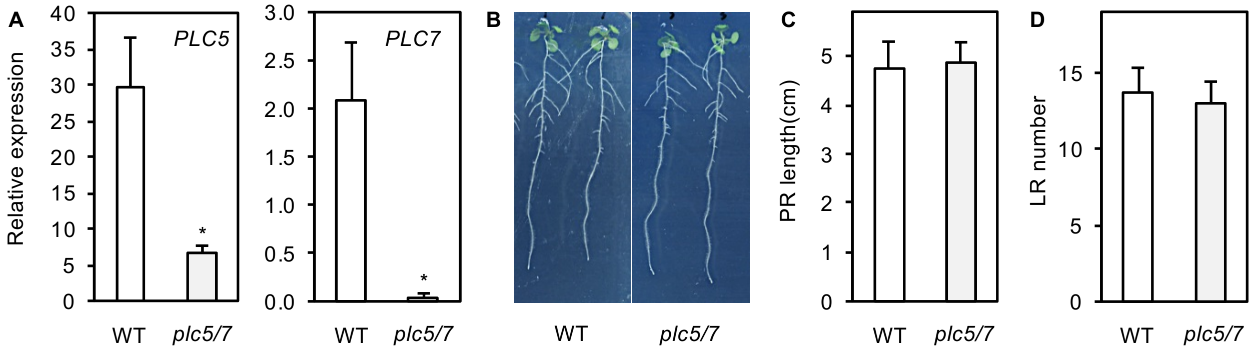

Supplemental figure S2 – Van Wijk *et al.*

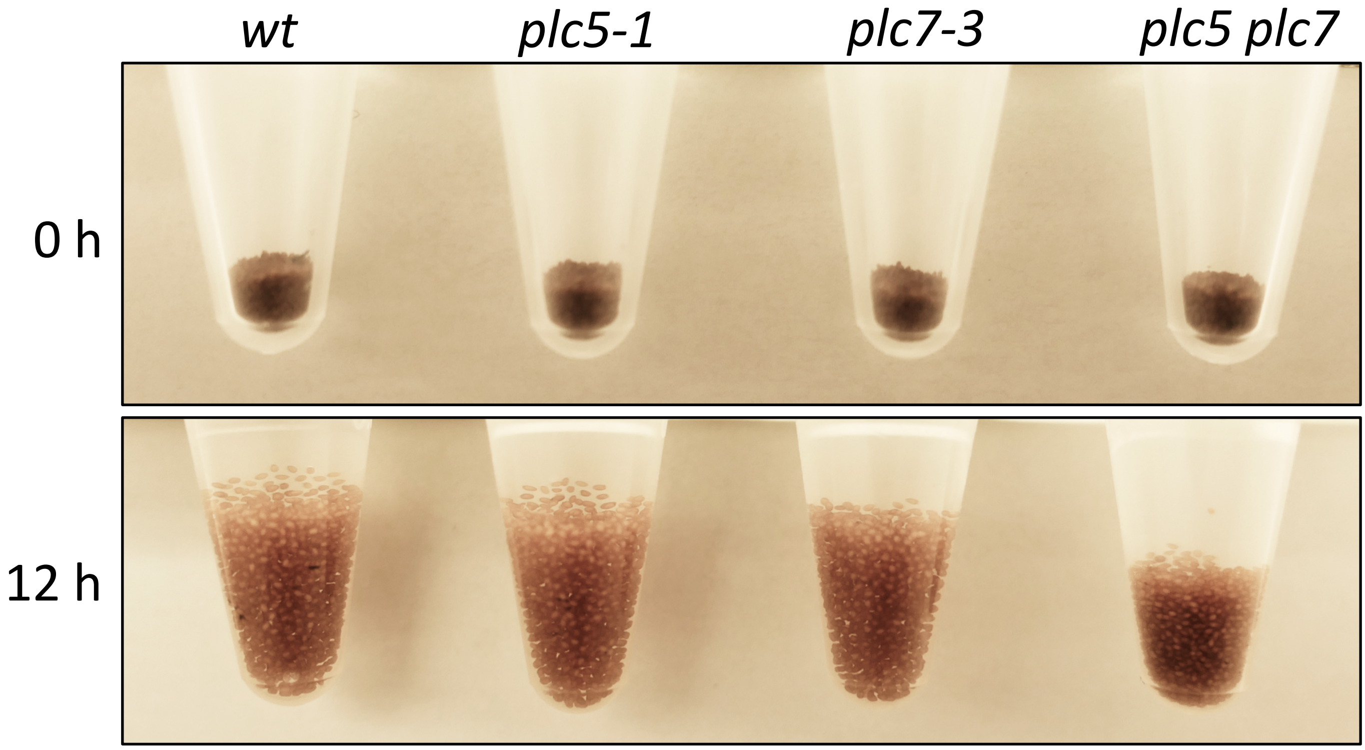

Supplemental figure S3 – Van Wijk *et al.*

A

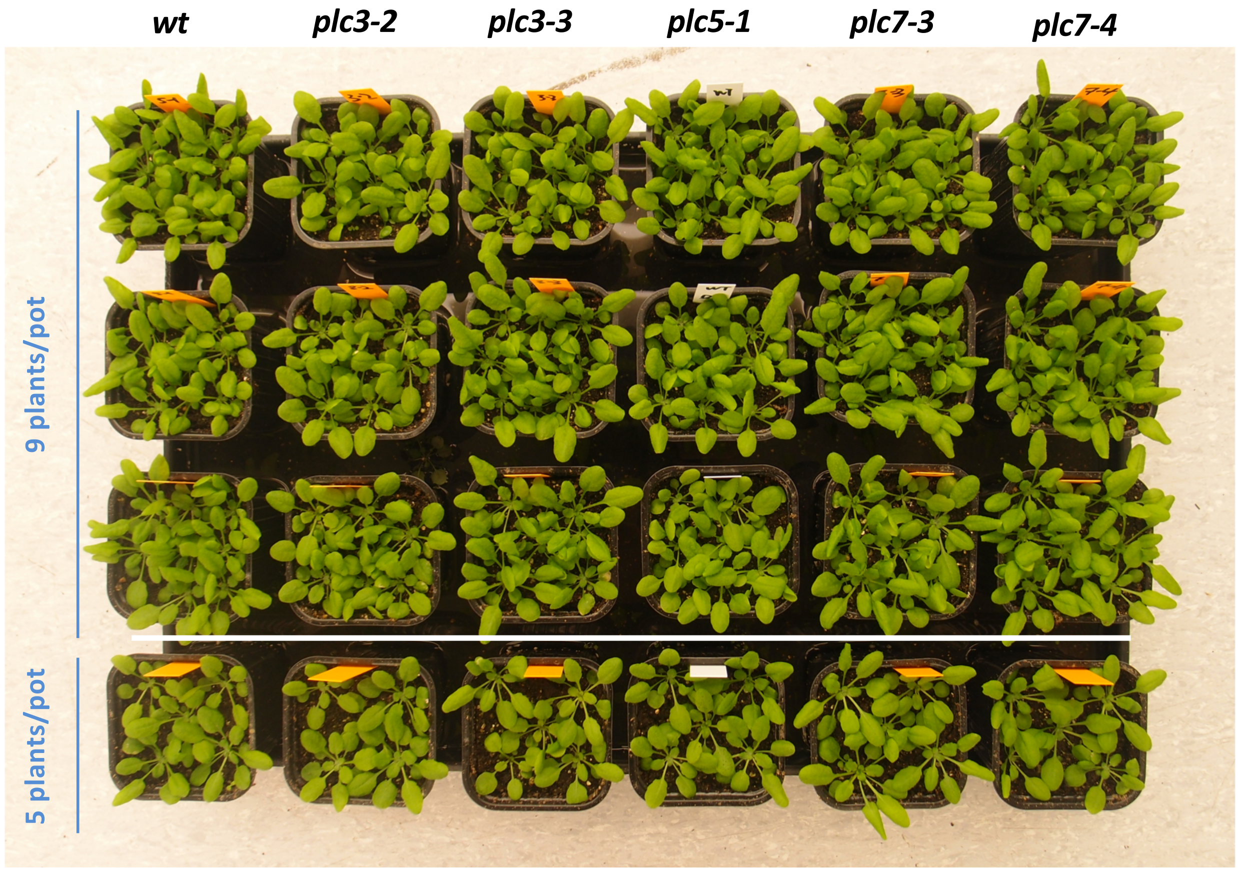

B

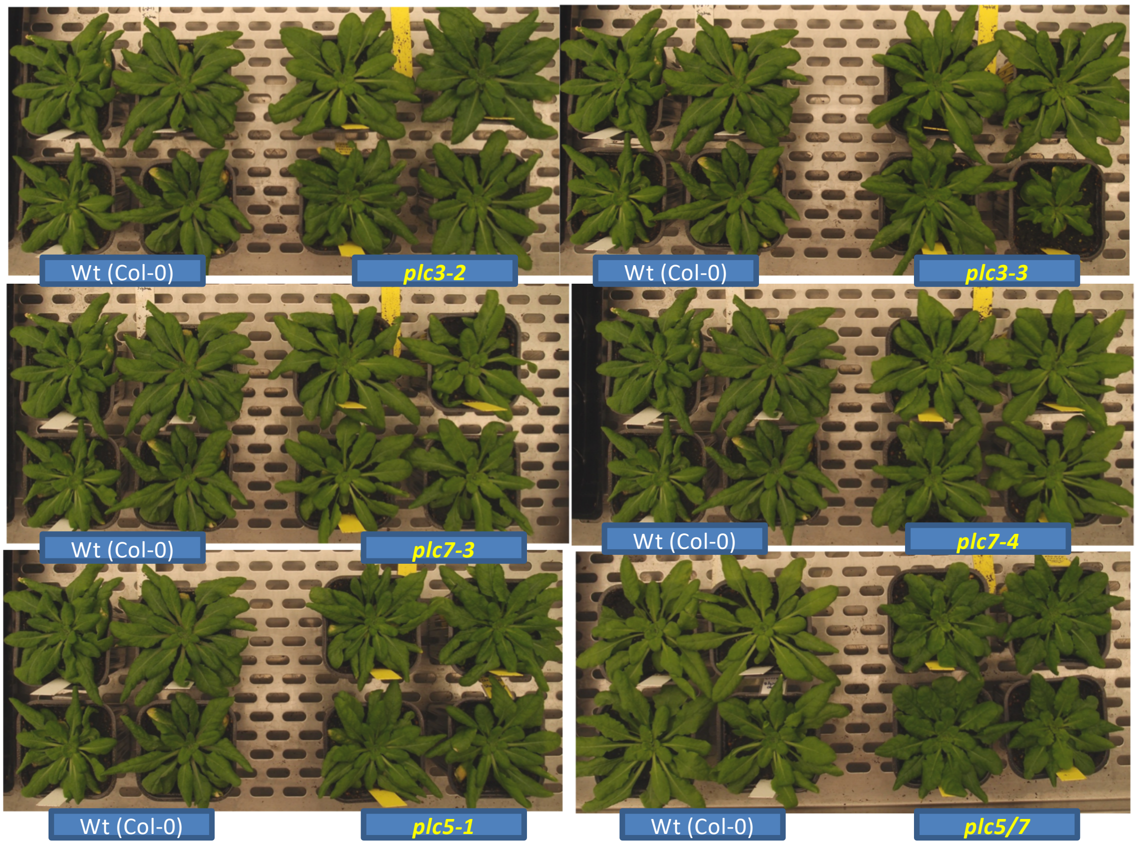

Supplemental figure S4 – Van Wijk *et al.*

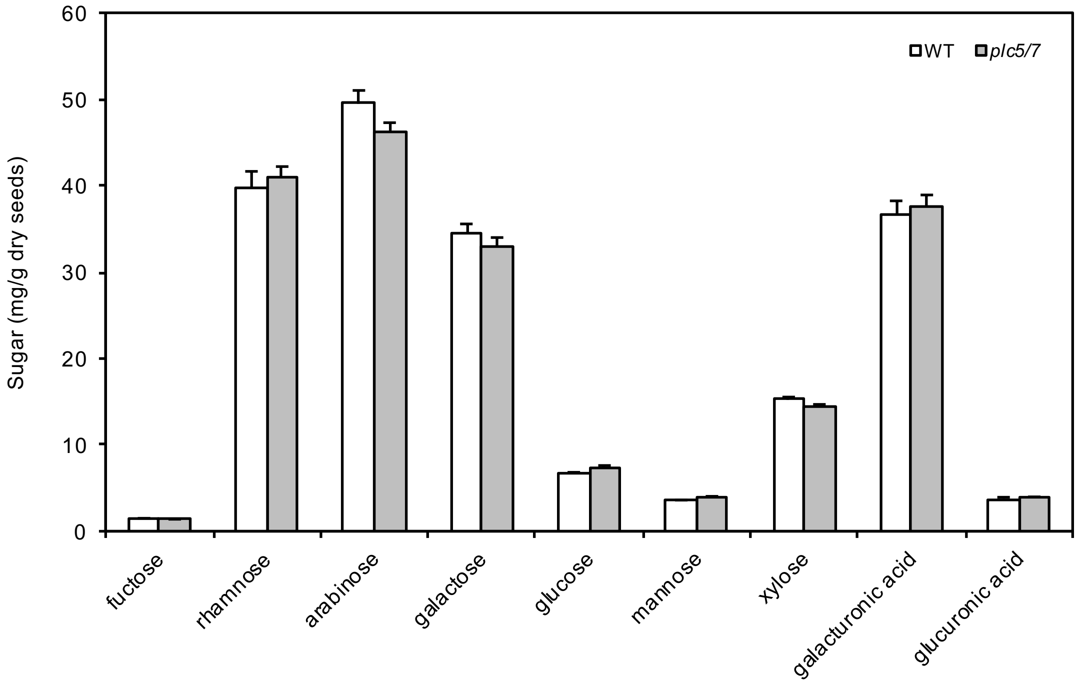

Supplemental figure S5 – Van Wijk *et al.*

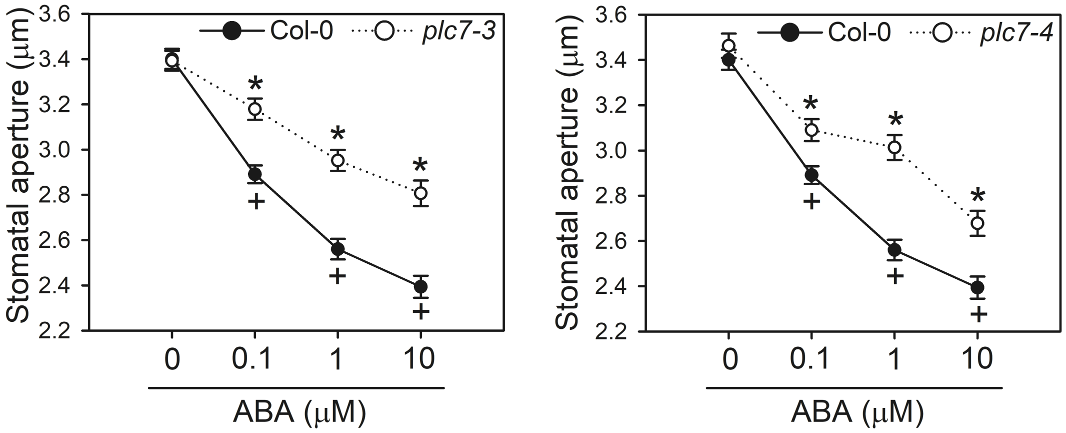

Supplement: Figure S1 — A plc5/plc7 double mutant is viable and not affected in root architecture. (A) Q-PCR expression levels of PLC5 (left) and PLC7 (right) in wild type and plc5/7-double mutant, using SAND as reference gene. Values are means ± SD (n = 3) of a representative experiment that was repeated three times. (B) Seedling morphology of wild type and plc5/7 (12 DAG). (C) Primary root (PR) length and (D) lateral root (LR) number at 12 DAG. Values are means ± SE of three independent experiments (n > 20). Asterisk (∗) indicate significance at P < 0.05 compared to wild-type, based on Student’s t-test. [file Data_Sheet_1.pdf]
